# A global database of abortion laws, policies, health standards and guidelines

**DOI:** 10.2471/BLT.17.197442

**Published:** 2017-06-09

**Authors:** Brooke Ronald Johnson, Vinod Mishra, Antonella Francheska Lavelanet, Rajat Khosla, Bela Ganatra

**Affiliations:** a Human Reproduction Programme, Department of Reproductive Health and Research, World Health Organization, 20 Avenue Appia, 1211 Geneva 27, Switzerland.; b Population Division, Department of Economic and Social Affairs, United Nations, New York, United States of America.

In June 2017 the United Nations Development Programme/United Nations Population Fund/United Nations Children’s Fund/World Health Organization (WHO)/World Bank Special Programme of Research, Development and Research Training in Human Reproduction (HRP) in collaboration with the Population Division of the United Nations Department of Economic and Social Affairs (UN DESA) launched a new, open-access Global Abortion Policies Database (available at two different web interfaces: http://www.srhr.org/abortion-policies; esa.un.org/gapp). The online database contains comprehensive information on the abortion laws, policies, health standards and guidelines for WHO and United Nations (UN) Member States. It is intended for use by policy-makers, human rights bodies, nongovernmental organizations, public health researchers and civil society.

The database is designed to further strengthen global and national efforts to eliminate unsafe abortion by facilitating comparative and country-specific analyses of abortion laws and policies, placing them in the context of information and recommendations from WHO technical and policy guidance on safe abortion.^1^^,^^2^ The main objectives of the database are to promote greater transparency of abortion laws and policies and State accountability for the protection of women and girls’ health and human rights.

## Backdrop to abortion policies

The association between restrictive abortion laws and unsafe abortion has been well documented.^3^^,^^4^ According to an analysis by UN DESA, the average rate of unsafe abortion is estimated to be more than four times higher in countries with more restrictive abortion laws than in countries with less restrictive laws.^5^ Restrictive abortion laws are also associated with higher levels of maternal mortality. The average maternal mortality ratio is three times higher in countries with more restrictive abortion laws (223 maternal deaths per 100 000 live births) compared to countries with less restrictive laws (77 maternal deaths per 100 000 live births).^5^ Restrictive legal grounds for abortion are only one of many policy barriers that affect women and girls’ access to safe abortion. Other barriers include policies that limit provision of abortion care to obstetricians and gynaecologists working at high-level care facilities; conscientious objection by health-care providers; requirements for third-party authorization(s); unnecessary medical tests; mandatory counselling; and mandatory waiting periods.^1^

Restrictive abortion laws and policies create risks to women and girls’ health by deterring them from seeking care and hindering providers from delivering services within the formal health system.^1^ Such laws and policies cause delays for women receiving care by creating complex and burdensome administrative procedures, increasing the costs of safe abortion services and limiting the availability of services and their equitable sociogeographic distribution.^1^ Such delays can also result in pregnancy advancing beyond legally allowed gestational limits, thus making women ineligible to receive safe services.^1^ Restrictions on access to safe abortion create inequalities both within and between countries, making access to safe abortion a privilege of the rich and leaving poor women little choice but to resort to illegal and usually unsafe practices and providers.^1^

## Consensus to eliminate unsafe abortion

WHO first recognized unsafe abortion as a serious public health problem in 1967.^6^ The problem was reaffirmed in the Programme of Action of the International Conference on Population and Development in 1994, which underscored the need for States to address the health consequences of unsafe abortion and to provide safe abortion where it is not against the law.^7^ Recognizing the public health challenge posed by unsafe abortion, WHO produced technical and policy guidance on safe abortion for health systems in 2003.^8^ A year later, prevention of unsafe abortion was recognized as a core component of the WHO *Reproductive health strategy to accelerate progress towards the attainment of international development goals and targets*.^9^ The strategy is grounded in international human rights treaties and global consensus declarations, such as the Programme of Action of the International Conference on Population and Development (Cairo, 1994) and the Fourth World Conference on Women (Beijing, 1995). In 2004, the WHO Member States endorsed the strategy.^9^^,^^10^ The WHO *Safe abortion: technical and policy guidance for health systems* was updated in 2012 and included a compilation of international human rights bodies’ observations on abortion laws and policies.^1^ Box 1 presents key policy recommendations from these guidelines.

Box 1 **WHO recommendations related to regulatory, policy and human rights considerations on abortion^1^**

- Laws and policies on abortion should protect women’s health and their human rights.
- Regulatory, policy and programmatic barriers that hinder access to and timely provision of safe abortion care should be removed.
- An enabling regulatory and policy environment is needed to ensure that every woman who is legally eligible has ready access to safe abortion care.
- Policies should be geared to respecting, protecting and fulfilling the human rights of women, to achieving positive health outcomes for women, to providing good-quality contraceptive information and services, and to meeting the particular needs of poor women, adolescents, rape survivors and women living with HIV.

HIV: human immunodeficiency virus; WHO: World Health Organization.

The guidelines also contain recommendations on methods, medical standards, service-delivery requirements for safe abortion, and provision of treatment for abortion complications.^1^ In 2015, emerging scientific evidence led to new WHO recommendations, which emphasized that safe abortion services in early pregnancy are primary-care-level procedures and specified which cadres of health workers can provide this care.^2^

## Improving information on abortion laws

Eliminating unsafe abortion will require multisectoral actions that create and strengthen enabling legal and policy environments for safe abortion and for the implementation of evidence-based policies and programmes aimed at improving service access and quality. However, to achieve these outcomes, the process must be informed by accurate and readily accessible information on countries’ existing laws and policies. Unfortunately, in many countries, policies that influence abortion access, availability and quality are difficult to obtain and use for decision-making, whether by women, girls, service providers or policy-makers.

Building on UN DESA’s World Population Policies Database, which has tracked legal grounds for abortion since the mid-1990s, the Global Abortion Policies Database aims to provide a more comprehensive information-resource tool. Initiated in early 2015, HRP engaged several research partners to retrieve abortion laws, policies, health standards and guidelines for WHO and UN Member States. Selected data were extracted onto a policy questionnaire that was rigorously cross-checked by public health and law experts and sent to countries for review.

The new database presents information on a broad range of policy domains: legal grounds and related gestational limits; authorization and service-delivery requirements; policies about who can provide abortion and where, when and how abortion services are permitted; and criminal penalties for women, girls, health-care providers and others (Box 2).

Box 2 **Policy domains highlighted in the Global Abortion Policies Database**

**Abortion legal grounds and related gestational age limits**

**Other legal requirements for abortion access**

- Third-party authorizations (parents, spouses, health-care providers, courts)
- Police reports for rape
- Compulsory counselling
- Mandatory waiting periods
- Medically unnecessary screening tests

**Who can be criminally charged and associated penalties for unlawful abortion**

**Restrictions on public information about abortion**

**National standards for abortion care**

- Existence of service-delivery guidelines
- Who can provide abortion services
- Where abortion can be provided
- Methods permitted
- National insurance coverage for abortion
- Registration of mifepristone and/or misoprostol

**Policies on conscientious objection**

In addition to data on specific abortion policies, individual country profiles include selected sexual and reproductive health indicators, links to State-ratified human rights treaties, and links to UN Treaty Monitoring Body Concluding Observations and Special Procedure Reports that address abortion. Most country abortion policies are juxtaposed to relevant information and recommendations from WHO *Safe abortion: technical and policy guidance for health systems*.^1^ All information in the database is linked to source documents that can be accessed and downloaded for further review.

The new database can help users to compare abortion laws and policies to the WHO guidelines, as well as among countries and geographical regions. It can also facilitate grasping the complexities and nuances of laws and policies that were not addressed previously or were obscured behind more simplistic classification schemes.

To promote greater transparency, the new database presents all abortion laws, policies and health standards as they are stated in the source documents. Guides on data collection and coding approach are available on the database websites. Extensive notes provide details on unique policy nuances and in those cases where multiple and sometimes conflicting policy documents exist.

The Global Abortion Policies Database has two important limitations: (i) some source documents are known to exist but were inaccessible to data collectors; and (ii) data extraction for source documents written in languages other than English was performed using unofficial translation tools when native speakers were not available. To mitigate these limitations, we encourage countries to engage with WHO/HRP and UN DESA/Population Division to ensure that data are accurate and source documents are current and complete.

Systematic updates of the database will be conducted every five years by formal engagement with countries through WHO country offices and health ministry counterparts. Interim updates will be made as needed, including when policies and laws are amended. Member States and other users are invited to provide suggestions for updates, corrections and feedback using the contact form available at the database websites. Interim updates will be posted periodically, pending receipt and verification of the primary source document(s).

## Conclusion

The global picture for abortion laws and policies is complex. Individual countries’ laws and policies can be protective or punitive, specific or non-specific, and limiting or facilitating for access and service provision. The new database provides a comprehensive compilation of country-specific documents and information on abortion in one readily accessible tool. This tool can facilitate increased transparency of available written laws and policies on abortion in a particular country. The database does not address how laws and policies are applied in practice, and so database users interested in progressive policy reform to protect women and girls’ health and human rights are encouraged to generate evidence on how laws and policies are implemented.
